# Pediatric Anaplastic Embryonal Rhabdomyosarcoma: Targeted Therapy Guided by Genetic Analysis and a Patient-Derived Xenograft Study

**DOI:** 10.3389/fonc.2017.00327

**Published:** 2018-01-11

**Authors:** Stuart L. Cramer, Aubrey L. Miller, Joseph G. Pressey, Tracy L. Gamblin, Elizabeth A. Beierle, Brian D. Kulbersh, Patrick L. Garcia, Leona N. Council, Rupa Radhakrishnan, Skyler V. Hendrix, David R. Kelly, Raymond G. Watts, Karina J. Yoon

**Affiliations:** ^1^Department of Pediatrics, University of Alabama at Birmingham, Birmingham, AL, United States; ^2^Department of Pharmacology and Toxicology, University of Alabama at Birmingham, Birmingham, AL, United States; ^3^Department of Surgery, University of Alabama at Birmingham, Birmingham, AL, United States; ^4^Department of Pathology, University of Alabama at Birmingham, Birmingham, AL, United States; ^5^The Birmingham Veterans Administration Medical Center, Birmingham, AL, United States; ^6^Department of Radiology, Cincinnati Children’s Hospital Medical Center, Cincinnati, OH, United States; ^7^Biomedical Science Program, UAB Honors College, University of Alabama at Birmingham, Birmingham, AL, United States; ^8^Department of Pathology and Laboratory Medicine, Children’s of Alabama, Birmingham, AL, United States

**Keywords:** anaplastic embryonal rhabdomyosarcoma, patient-derived xenograft, case study, vorinostat, targeted therapy

## Abstract

Therapy for rhabdomyosarcoma (RMS) has generally been limited to combinations of conventional cytotoxic agents similar to regimens originally developed in the late 1960s. Recently, identification of molecular alterations through next-generation sequencing of individual tumor specimens has facilitated the use of more targeted therapeutic approaches for various malignancies. Such targeted therapies have revolutionized treatment for some cancer types. However, malignancies common in children, thus far, have been less amenable to such targeted therapies. This report describes the clinical course of an 8-year-old female with embryonal RMS having anaplastic features. This patient experienced multiple relapses after receiving various established and experimental therapies. Genomic testing of this RMS subtype revealed mutations in *BCOR, ARID1A*, and *SETD2* genes, each of which contributes to epigenetic regulation and interacts with or modifies the activity of histone deacetylases (HDAC). Based on these findings, the patient was treated with the HDAC inhibitor vorinostat as a single agent. The tumor responded transiently followed by subsequent disease progression. We also examined the efficacy of vorinostat in a patient-derived xenograft (PDX) model developed using tumor tissue obtained from the patient’s most recent tumor resection. The antitumor activity of vorinostat observed with the PDX model reflected clinical observations in that obvious areas of tumor necrosis were evident following exposure to vorinostat. Histologic sections of tumors harvested from PDX tumor-bearing mice treated with vorinostat demonstrated induction of necrosis by this agent. We propose that the evaluation of clinical efficacy in this type of preclinical model merits further evaluation to determine if PDX models predict tumor sensitivity to specific agents and/or combination therapies.

## Introduction

Rhabdomyosarcoma (RMS) is the most common soft-tissue sarcoma of childhood. This tumor type is characterized by myoblastic differentiation and expression of skeletal muscle markers such as desmin, myogenin, and/or MYOD1. Embryonal RMS (eRMS), the most common subtype, usually occurs before 10 years of age. A secondary subtype, anaplastic eRMS, is characterized by enlarged hyperchromatic nuclei and TP53 mutations, and is associated with poor outcome (1, 2). Chemotherapy, surgery, and/or radiation comprise standard therapy for patients with RMS. Chemotherapeutic regimens frequently include vincristine, actinomycin D, cyclophosphamide, and inhibitors of type-1 topoisomerase or mammalian target of rapamycin (mTOR) (3, 4).

Here, we report the case of a patient who presented with an anaplastic eRMS of the left parapharyngeal pterygopalatine fossa. At diagnosis, the tumor mass measured 3.9 cm × 3.1 cm × 3.6 cm. The patient’s treatment history included multiple chemotherapeutic regimens, palliative radiation (XRT), and debulking procedures (Table 1). Responses were transient, and several localized relapses were documented during the 4 years of treatment. Tissue from final surgical resection on 4/22/2014 was used to establish the patient-derived xenograft (PDX) model described in this study and to submit for sequencing (Foundation Medicine, Cambridge, MA, USA). Sequence analysis identified mutations in genes encoding *BCOR, ARID1A*, and *SETD2* (Table 2). These mutations would be predicted to increase histone deacetylases (HDAC) activity or confer gain of function or constitutive activation of HDAC. Therefore, the patient was treated with the HDAC inhibitor vorinostat for 6 months.

**Table 1:** Summary of relevant clinical history.

Date	Treatment received
8/2010	Vincristine, actinomycin, and cyclophosphamide (VAC) with XRT^a^ therapy
4/2012	VDC^b^/IE^c^ with Gamma Knife
9/2013	Vinorelbine, temsirolimus, and cyclophosphamide
11/2013	Crizotinib
12/2013	Cabozantinib
2/2014	Eribulin
4/2014	MK1775
4/22/2014	Debulking procedure, tracheostomy placement, XRT (acquisition of tumor specimen for DNA sequencing and PDX development)
6/2014	Gemcitabine and docetaxel
8/2014	Vorinostat
1/2015	Neck swelling observed
2/2015	Vorinostat discontinued
4/2015	Death of the patient

*^a^Radiation therapy*.

*^b^Vincristine, doxorubicin, and cyclophosphamide*.

*^c^Ifosfamide and etoposide*.

**Table 2:** Genetic alterations identified by sequencing.

Gene	Mutations
*ARID1A*	D1850fs*4 M1634fs*1
*BCOR*	R546fs*16
*SETD2*	S2382fs*47 T2513fs*4

Treatment with vorinostat was initiated 4 months after surgical resection in April 2014. Tumor specimens obtained prior to administration of vorinostat provided the opportunity to establish a PDX model from this patient’s tumor, and to use this model to determine if preclinical data characterizing the efficacy of vorinostat reflected the efficacy of this agent in the clinic. A goal of the study was to evaluate whether this type of model might be used to predict efficacy in the clinic, as an approach toward personalized medicine.

## Background

There are no published studies describing the utility of vorinostat in treating RMS, but several preclinical studies have been published. Keshelava et al. demonstrated that vorinostat had IC_50_ values of 0.88–9.77 µM in four RMS cell lines (RD, Rh41, Rh18, and Rh30) *in vitro*, but had little or no effect in five RMS xenograft models (Rh30, Rh30R, Rh41, Rh18, and Rh36) (5). A second study by Vleeshouwer-Neumann et al. reported that vorinostat suppressed the growth of RD, 381 T, and SMS–CTR eRMS cell lines and also inhibited the migration of these cells *in vitro* (6). Furthermore, these investigators observed that vorinostat had antiproliferative effects in a zebrafish transgenic eRMS model.

### Tumor DNA Sequencing

Sequencing of the 400 genes in the Pediatric Cancer Prone Gene Panel was performed by Foundation One (CLIA Certified Sequencing Foundation, Cambridge, MA, USA) using DNA extracted from formalin-fixed paraffin-embedded (FFPE) tumor tissue. Mutations were identified in genes encoding *BCOR, ARID1A*, and *SETD2*, which are involved in DNA methylation and chromatin remodeling and each of which affects HDAC-associated cell processes (7–9).

### *BCOR* 

*BCOR* (BCL6 corepressor; Polycomb group repressive complex-1 variant) mediates BCL6 function in diffuse large B-cell lymphoma (10, 11). *BCOR* inhibits histone methylation (H3 Lys-4:H3K4me3 and Lys-36:H3K36me2), an activity regulated by HDAC (7, 12). Studies with mesenchymal stem cells obtained from patients with oculofacialcardiodental syndrome (OFDC) indicate that mutations of *BCOR* enhance mesenchymal stem cell proliferation (7). Mutations in this gene are also associated with myelodysplastic syndromes, pediatric acute myeloid leukemia, and medulloblastoma (13–15).

### *ARID1A* 

*ARID1A* (AT-rich interaction domain 1A) is a member of the SWI/SNF family and has helicase and ATPase activities (16). ARID1A regulates transcription by altering the structure of chromatin (8). ARID1A also contributes to regulation of cell-cycle progression and is involved in DNA damage repair (17, 18). Mutations in *ARID1A* have been identified in ovarian, endometrial, and uterine tumors (19–22). Inactivating mutations in *ARID1A* suggest that wild-type *ARID1A* may act as a tumor suppressor (23). *In vitro* studies indicate that ovarian cancer cells harboring *ARID1A* mutations are relatively sensitive to the histone methyltransferase EZH2 inhibitor and to nutlin, which inhibits MDM2–p53 interaction (24, 25).

### *SETD2* 

*SETD2* (SET domain containing 2) is a histone methyl transferase that methylates Lys36 of histone H3 (H3K36Me3) (9, 26). Loss-of-function mutations of this gene promote renal cancer progression and decrease expression of H3K36Me3 in clear cell renal cell carcinoma (27). SETD2–H3K36Me3 pathway alterations are associated with development of leukemia (28).

### HDAC

Histone deacetylases regulate the ratio of acetylated and deacetylated histones, and are associated with gene silencing by modification of chromatin structure (29, 30). Alterations in *HDAC* genes and/or HDAC expression are related to multiple human pathologies including cancer (31–35). For example, increased HDAC1 and HDAC2 expression is reported in colon cancer cells compared with non-oncogenic adenoma cells, and decreased expression of HDAC1 arrests the growth of this tumor cell type (36, 37).

Each of the epigenetic alterations described above has been reported to contribute to tumor phenotypes (9, 13, 15, 27, 28, 38, 39), and each of the three proteins encoded by genes identified as harboring mutations contributes directly or indirectly to HDAC-associated functions. The HDAC inhibitor vorinostat (suberoylanilide hydroxamic acid, SAHA) is approved for refractory T-cell lymphoma and is being evaluated in multiple clinical trials for other types of cancers (40). Based on the patient’s tumor characteristics, the FDA-approved status of vorinostat, the known maximum tolerated dose in pediatric patients, and preclinical information in the literature, the patient was treated with vorinostat.

### Vorinostat (Suberoylanilide Hydroxamic Acid, an HDAC Inhibitor)

Vorinostat was approved in the USA in 2006 for the treatment of cutaneous T-cell lymphoma (CTCL) in patients with progressive or recurrent disease (41). A Phase-I COG clinical trial demonstrated that vorinostat was well tolerated at 230 mg/m^2^/day in children with recurrent solid tumors (42). Vorinostat inhibits the activity of HDACs 1 and 2, thereby increasing the ratio of deactylated/acetylated histones and suppressing tumor cell proliferation *in vitro* and *in vivo*. Current literature suggests that the likely mechanism of this suppression is that HDAC deacetylation selectively activates transcription of genes that induce cell differentiation and/or apoptosis (43, 44).

## Discussion

### Ethics Statement

Protocols involving human subjects were approved by the Institutional Review Board (IRB) of the University of Alabama at Birmingham (Birmingham, AL, USA). Written informed assent and consent were obtained from the patient and her family to use tumor specimens for research purposes. Written informed consent was also obtained from the patient’s parent agreeing to publication of the report.

### Clinical Course Associated with Vorinostat Treatment

**Figure 1:**
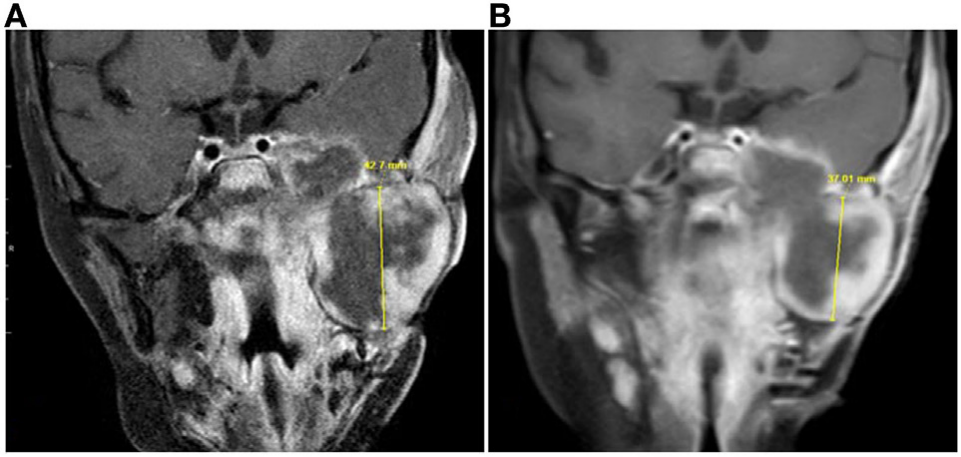
Magnetic resonance imaging (MRI) taken prior to and during vorinostat treatment. Coronal contrast-enhanced T1-weighted MRI images from **(A)** July 2014 and **(B)** September 2014 showed decrease in size of the infratemporal component of the tumor. Craniocaudal measurement showed the greatest change, although this component of the tumor had decreased in size in other dimensions as well.

Treatment consisted of 28-day cycles at 200 mg/day (42). Approximately 4 weeks after the initiation of treatment, magnetic resonance imaging (MRI) of brain, orbit, and neck revealed a reduction of the tumor mass centered at the left infratemporal fossa from 8.9 cm × 4.9 cm × 6.5 cm to 8.2 cm × 4.7 × 6.0 cm (Figure 1). The dose of vorinostat was increased to 300 mg/day 3 days/week and 200 mg/day 4 days/week. Within 2 weeks of dose escalation, the patient developed severe thrombocytopenia associated with nasopharyngeal hemorrhage requiring packed red blood cell resuscitation. The dose was then de-escalated to 200 mg/day. Over the next 16 weeks, MRIs documented stabilization of the infratemporal fossa tumor mass, with sustained necrosis. While receiving vorinostat, the patient showed symptomatic improvement and was weaned from patient-controlled analgesia. Following this transient response, the patient developed neck swelling due to tumor progression and tumor rupture traversing the skin. Two months after the 6-month regimen of single-agent vorinostat had been discontinued, the patient succumbed to her disease.

### Response to Vorinostat of Mice Bearing Patient-Derived Xenografts

#### Ethics Statement

Protocols involving animal use were approved by the Institutional Animal Care and Use Committee (IACUC) of the University of Alabama at Birmingham (Birmingham, AL, USA).

A tumor specimen harvested 4 months prior to initiating vorinostat therapy was implanted subcutaneously into immunocompromised mice (SCID) within 1 h of tumor resection. When the tumor volume of the donor mouse reached ~800–1,000 mm^3^, the tumor was harvested, divided, and transplanted into a cohort of mice for evaluation of vorinostat efficacy. When tumor volume reached ~300 mm^3^, tumor-bearing mice were randomized into two groups (*N* = 10/group) and received 50 mg/kg vorinostat or vehicle (vehicle control) intraperitoneally daily for 21 days. This dose is equivalent to a clinical dose of ~200 mg (45). Tumors were measured with Vernier calipers (Fowler/Slyvac, Newtown, MA, USA) twice weekly, and tumor volumes calculated using the equation *v* = (π/6)d^3^. Twenty-four hours after completion of treatment, mice were euthanized, and tumor tissue was harvested and archived as both formalin fixed paraffin embedded (FFPE) and snap frozen in liquid nitrogen. Tumor volumes were compared by two-way analysis of variance (ANOVA) followed by Bonferroni posttest (GraphPad Prism 5.0). Values presented equal mean ± SEM.

**Figure 2:**
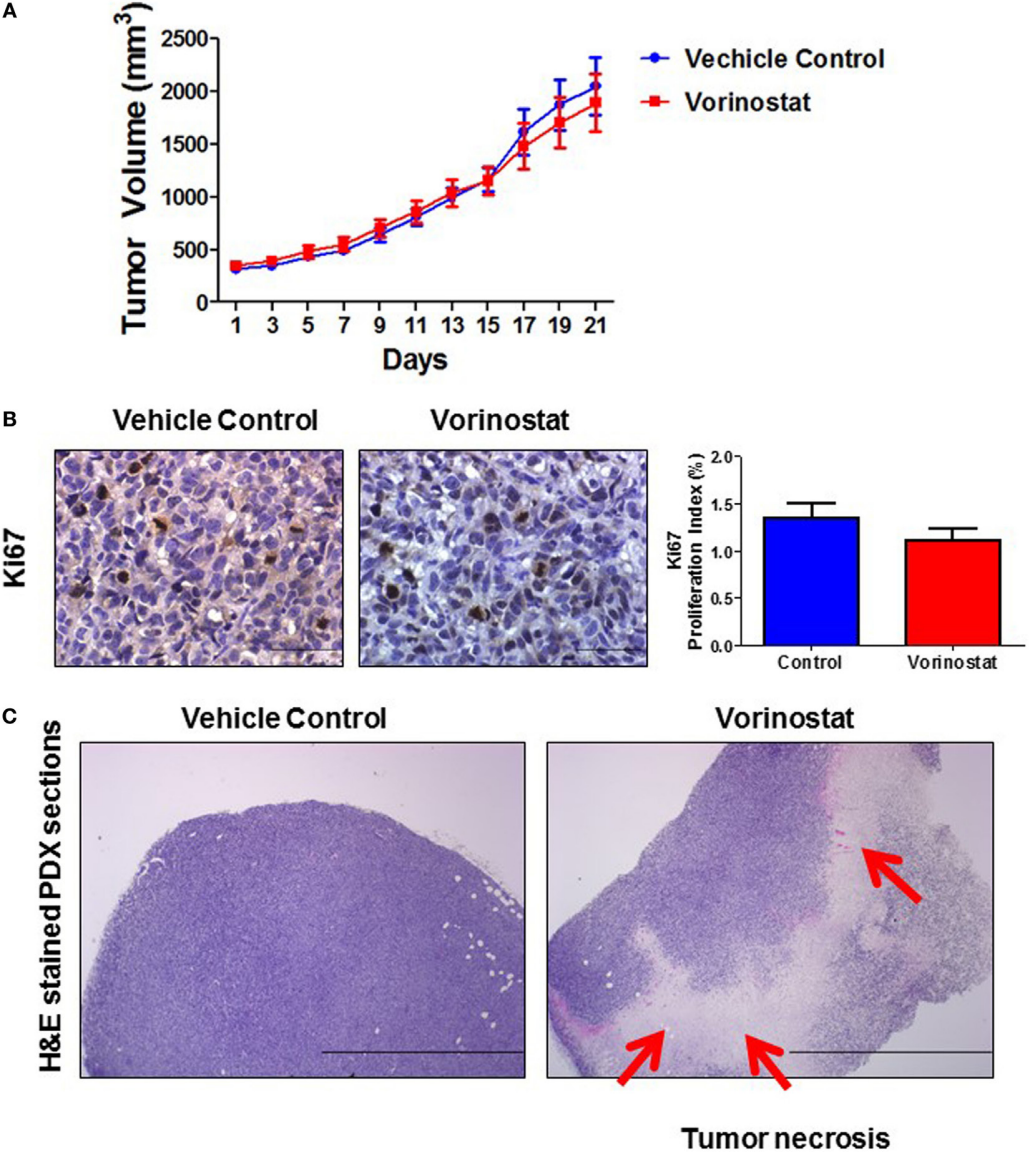
Monotherapy of vorinostat which did not suppress tumor growth in a patient-derived xenograft model (COA/UAB-15), but did induce tumor necrosis. **(A)** When tumor volumes reached ~300 mm^3^, 50 mg/kg vorinostat or vehicle was administered once a day to tumor-bearing mice (*N* = 10/group) for 21 days. **(B)** Immunohistological analysis for the proliferation marker Ki67 showed no difference in growth fraction between vorinostat- and vehicle-treated mice group. **(C)** H&E staining of formalin-fixed paraffin-embedded tissue prepared from tumor harvested from vehicle-treated or vorinostat-treated mice 24 h after the last day of treatment. Red arrows indicate examples of necrotic areas.

As shown in Figure 2A, the 21-day treatment of vorinostat did not inhibit anaplastic eRMS tumor growth in the PDX model. Immunostaining for the proliferation marker Ki67 (Figure 2B) showed no difference between drug- and vehicle-treated groups. However, interestingly, vorinostat-treated tumors had obvious necrotic regions compared with vehicle controls, as determined by histopathologic analysis (LNC; Figure 2C). Although there were no differences in tumor volumes between vorinostat-treated and vehicle control-treated groups by the end of the 21-day treatment study, we observed that vorinostat-treated mice had “softened” tumors starting on day 7, which we regarded as consequent to drug treatment (Figure 2C) and which may correspond with the necrosis observed by MRI imaging of the primary tumor *in situ* (Figure 1).

## Concluding Remarks

In this study, we report evaluation of the efficacy of vorinostat, a non-standard treatment, for a patient with anaplastic eRMS and in a parallel preclinical study using a PDX model developed from resected tumor tissue. The patient was treated with the HDAC inhibitor vorinostat, based on the mutational status of her recurrent chemorefractory tumor. Clinically, vorinostat treatment induced a transient tumor regression, followed by tumor progression (increase in tumor volume at primary site). Necrotic areas of the tumor following vorinostat treatment were documented by MRI (Figure 1). The preclinical evaluation of vorinostat efficacy using the PDX model reflected clinical observations with respect to induction of tumor necrosis. Notably, this is the first report demonstrating that vorinostat induces necrosis *in vivo* (Figure 2C). We propose that more successful treatment for solid tumors lies in understanding the molecular and genetic characteristics that confer specific malignant phenotypes, and in the use of well-characterized preclinical models for evaluating novel agents with potential efficacy.

## Ethics Statement

Ethics statement: Protocols involving human subjects were approved by the Institutional Review Board (IRB) of the University of Alabama at Birmingham (Birmingham, AL, USA). Written informed assent and consent were obtained from the patient and her family to use tumor specimens for research purposes. Written informed consent was also obtained from the patient’s parent agreeing to publication of the report. Protocols involving animal use were approved by the Institutional Animal Care and Use Committee (IACUC) of the University of Alabama at Birmingham (Birmingham, AL, USA).

## Author Contributions

SC, JP, and KY contributed to the conception and design of the study. AM, TG, PG, and SH contributed to the acquisition of data (coordinate specimen acquisition and performed experiments). SC, JP, AM, LC, RR, and DK contributed to the analysis and interpretation of data. SC, JP, and KY contributed to the writing of the manuscript. BK and EB contributed to the clinical resection. RW contributed to the administrative or material support. SC and KY contributed to the study supervision. All authors contributed to review and revision of the manuscript.

## Conflict of Interest Statement

The authors declare that the research was conducted in the absence of any commercial or financial relationships that could be construed as a potential conflict of interest.
